# Trust but verify: Is there a role for active surveillance in monitoring adverse events in Zimbabwe’s large-scale male circumcision program?

**DOI:** 10.1371/journal.pone.0218137

**Published:** 2019-06-10

**Authors:** Phiona Marongwe, Paidamoyo Gonouya, Thoko Madoda, Vernon Murenje, Mufuta Tshimanga, Shirish Balachandra, John Mandisarisa, Vuyelwa Sidile-Chitimbire, Sinokuthemba Xaba, Batsirai Makunike-Chikwinya, Marrianne Holec, Scott Barnhart, Caryl Feldacker

**Affiliations:** 1 International Training and Education Center for Health (I-TECH), Harare, Zimbabwe; 2 Zimbabwe Association of Church-related Hospitals (ZACH), Harare, Zimbabwe; 3 Zimbabwe Community Health Intervention Project (ZiCHIRe), Harare, Zimbabwe; 4 U.S. Centers for Disease Control and Prevention, Harare, Zimbabwe; 5 Ministry of Health and Child Care, Harare, Zimbabwe; 6 International Training and Education Center for Health (I-TECH), Seattle, WA, United States of America; 7 Department of Medicine, University of Washington, Seattle, WA, United States of America; 8 Department of Global Health, University of Washington, Seattle, WA, United States of America; Makerere University School of Public Health, UGANDA

## Abstract

**Introduction:**

Ensuring quality service provision is fundamental to ZAZIC’s voluntary medical male circumcision (MC) program in Zimbabwe. From October, 2014 to September, 2017, ZAZIC conducted 205,847 MCs. Passive surveillance recorded a combined moderate and severe adverse event (AE) rate of 0.3%; reported adherence to follow-up was 95%, suggesting program safety. Despite encouraging passive surveillance data, verification of data quality and accuracy would increase confidence in AE identification.

**Methods:**

From May to August, 2017, ZAZIC implemented a focused quality assurance (QA) study on AE ascertainment and documentation at 6 purposively-selected, high-volume MC sites. ZAZIC Gold-Standard (GS) clinicians prospectively observed 100 post-MC follow-ups per site in tandem with facility-based MC providers to confirm and characterize AEs, providing mentoring in AE management when needed. GS clinicians also retrospectively reviewed site-based, routine MC data, comparing recorded to reported AEs, and held brief qualitative interviews with site leadership on AE-related issues.

**Results:**

Observed AE rates varied from 1–8%, potentially translating to thousands of unidentified AEs if observed AE rates were applied to previous MC performance. Most observed AEs were infections among younger clients. Retrospective review found discrepancies in AE documentation and reporting. Interviews suggest human resource and transport issues challenge MC follow-up visit attendance. Post-operative self-care appears to produce generally good results for adults; however, younger clients and guardians need additional attention to ensure quality care. There was no evidence of missed severe AEs resulting in permanent impairment or morbidity.

**Conclusions:**

Although results cannot be generalized, active surveillance suggests that AEs may be higher and follow-up lower than reported. In response, ZAZIC’s Quality Assurance Task Force will replicate this QA study in other sites; increase training in AE identification, management, and documentation for clinical and data teams; and improve post-operative counseling for younger clients. Additional nurses and vehicles, especially in rural health clinics, could be beneficial.

## Introduction

Accurate, timely, and reliable public health data are essential for the delivery of high-quality healthcare services. In voluntary medical male circumcision (MC) programs, a key indicator of program quality is the rate of adverse events (AEs)[1]. AEs in clinical MC settings are uncommon with few mortalities [2]. After clinical trials in sub-Saharan Africa demonstrated that MC was safe [3–5], an AE rate of 2% moderate or severe AEs became a commonly-used standard for safety [6, 7]. In general, *severe* AEs are those requiring surgical intervention or hospitalization, whereas any AE not classified as severe, but which requires intervention by a health care provider or medication, is considered *moderate* [8, 9]. AEs are also categorized by type, e.g. infection, bleeding, etc. [9]. AE rates may be higher in MC programs operating at scale without the clinical oversight and control of research studies. Although AE reporting definitions may differ slightly, field settings with active surveillance (proactive patient follow-up) often report higher AE rates, varying from 7% [10, 11] to nearly 18% [12]. In contrast, passive surveillance settings (typically routine care settings without added proactive patient tracing) rely predominantly on clients presenting at a health facility. AE rates from passive surveillance in sub-Saharan Africa report lower AE rates, beneath the 2.0% threshold [13, 14]. In countries and settings with severe healthcare shortages and fewer resources, including Zimbabwe [15], passive surveillance may be weak [16].

Lack of reliable, quality data jeopardizes program integrity and, potentially, patient safety. Between 2008 and 2016, Zimbabwe’s Ministry of Health and Child Care (MoHCC) reported 807,060 MCs [17] and a national AE rate of 0.35%, an estimated 2,825 AEs [18]. However, using the global standard of 2% AEs, Zimbabwe should have approximately 16,141 complications. AE identification, documentation and reporting among MC programs in sub-Saharan Africa may be affected by poor data quality [19–21]. In Zimbabwe, previous studies on data quality and AE identification within the MC program revealed challenges to correct and complete data [22, 23]. The low AE rate in Zimbabwe may indicate potential AE underreporting.

ZAZIC, named for the partners that formed a consortium in 2013 (International Training and Education Center for Health (I-TECH), Zimbabwe Association of Church related Hospitals (ZACH) and Zimbabwe Community Health Intervention Research Project (ZiCHIRe)), cooperates with Zimbabwe’s MoHCC to implement a large-scale, integrated MC program across 10 districts [24]. Ensuring quality service provision is a critical component of ZAZIC’s MC program. ZAZIC implements routine monitoring and evaluation (M&E) activities to identify and address weaknesses in data collection, data reporting, and data use. From October, 2014 to September, 2017, ZAZIC conducted 205,847 MCs [25]. Over this highly productive period, the reported moderate and severe AE rate from the routine, passive surveillance system was 0.3% [13, 24, 26], a rate far lower than the global standard. ZAZIC’s reported adherence to the standard indicator of “at least one follow-up visit within 14 days of MC,” another indicator of program quality [27], was high at 95% (P. Marongwe, personal communication, May 31, 2018). However, as with other passive surveillance programs, ZAZIC was concerned that the low rate of reported AEs could reflect potential underreporting.

Therefore, to review AE data and confirm the reported AE rate, ZAZIC conducted a quality assurance (QA) study in 2017. The study had two objectives: 1) to strengthen MC program AE documentation and 2) to increase confidence in the quality of reported AE data. The mixed-method study took place from May, 2017 to August, 2017 and included three components: 1) 100 prospective tandem MC reviews with a gold-standard (GS) ZAZIC clinician working alongside site-based, MoHCC MC clinicians; 2) retrospective review and comparison of three previous months of routine AE data from MC registers, client intake forms (CIF), monthly return forms (MRF) and District and Health Information Systems (DHIS2); and 3) brief qualitative interviews with MoHCC site staff about AE-related practices. GS clinicians provided in-person mentoring, support, and feedback on the results of both the prospective and retrospective activities before concluding site visits. It is hoped these results will improve confidence in ZAZIC’s AE data and spur similar QA efforts across other MC programs operating at scale.

## Methods

### Site selection

Six high-volume MC sites that each conducted more than 5000 MCs, annually, were purposively selected (one urban and 5 rural). All sites started offering MC in 2014. At all six high-volume sites, MCs were conducted at static and outreach sites, including at Rural Health Centers (RHC). High-volume sites were selected to help ensure observation of AEs in consideration of the low AE rates. The majority of district MCs were performed by static site teams who traveled to outreach sites (schools, RHCs, tents in central locations, etc.). These MC teams are not dedicated staff for MC services; rather, MC is offered by MoHCC providers. Follow up care for clients is provided by both the MC team from the static site and RHC nurses. As reported program AEs are low, a target of 100 tandem reviews was set at each high-volume site to potentially identify at least 1 AE within a reasonable observation time frame in consideration of routine constraints on GS time.

### Selection and training of “Gold Standard” (GS) staff

The ZAZIC GS team was comprised of 3 clinicians, two nurses and one doctor, with expertise in MC provision, including documentation and management of AEs. All GS clinicians were also trainers for the national MC program, and, as such, received training by specialist surgeons and urologists in the MC program. GS-specific training for this study included group review of AE severity guidelines, management protocols, and reporting requirements using World Health Organization (WHO) AE guidelines [28]. GS team reviewed recently reported AEs to help ensure inter-rater reliability (AE type, code, and severity) in the field. The GS team practiced record review at one non-selected site to reach consensus on acceptable data quality standards.

### Site routine MC program procedures

All ZAZIC sites and MC procedures follow MoHCC guidelines and World Health Organization (WHO) standards for quality MC service provision, demand creation, and data management [28–32], as described previously [24]. In brief, all clients undergo MC counseling, clinical review, HIV counseling and testing, and informed consent before the MC procedure. Boys ages 10 and older are eligible for MC using the dorsal slit method; males age 15 and older may be circumcised using either dorsal slit or forceps guided methods. Post-operative checks and wound care counseling are conducted before releasing clients. Standard MC follow-up includes scheduling of routine visits for all surgical MC clients on post-operative days 2, 7, and 42. Follow-up occurs most commonly at the health facilities where MCs took place. If clients fail to attend days 2 or 7, MoHCC policies recommend active follow-up by phone or home visit. In general, MC nurses or clerks record client information, including follow-up and AEs, into MC registers and CIFs. In addition, an MoHCC AE notification form is used to report moderate and severe AEs to MoHCC’s provincial office and to ZAZIC. Data from the register are aggregated (clients ages, MC method, AE, follow-up attendance) into the monthly return form (MRF) and submitted for DHIS2. Site teams also submit monthly aggregate data on a ZAZIC-specific reporting form that contains additional details on AEs. All ZAZIC districts have at least one district or mission hospital and satellite outreach sites offering MC. Each static site has one dedicated program vehicle to support all MC program activities. At the time of the study, the MoHCC had suspended implementation of device-based MCs; therefore, only surgical MCs were conducted during the time of this review.

### Study-specific procedures

The study was implemented between May, 2017 and August, 2017. For the prospective tandem reviews, component one, each of the three GS clinician conducted tandem reviews in two sites, primarily from Tuesdays to Thursdays. Within the May to August, 2017, period, the specific week chosen for GS site-team attachment was based on at least 50 MCs the previous week and GS availability. If consecutive weeks were not similarly high volume, GS clinicians returned when productivity increased. At MoHCC sites, the matron or MC focal person routinely assigned, daily or weekly, one MC clinician (most commonly, a nurse) to conduct all scheduled and non-routine reviews. At each site, GS teams in tandem with the one MoHCC clinician first reviewed all clients at the static site and then scheduled client follow-up from outreach sites or active tracing lists. For each client, the GS observed the MoHCC clinician’s post-operative review, confirmed presence or absence of AEs, determined AE type and severity (when applicable), and reviewed documentation. If needed, the GS accompanied a client for secondary review by another MoHCC clinician, where another GS tandem review was completed. The second study component, also performed at each site, was a retrospective record review of data collected for the 3-month period, October, 2016 to December, 2016. The three-month period October, 2016 to December, 2016 was chosen as it was not included in the previous data quality audit and, therefore, was considered to reflect data that had not been previously reviewed or corrected. While at each static site, the GS gathered, reviewed and compared the AEs recorded on CIFs, MC register, MoHCC AE forms, out- or in-patient hospital registers, or other ad hoc AE forms between October and December, 2016. The third component was also conducted while at each site. The GS engaged MC team members in a brief qualitative activity: a discussion of findings from both the prospective reviews and the retrospective review. The GS, themselves, completed a brief, paper-based questionnaire on AE recognition, reporting and management based on the conversation. GS attachments lasted approximately one week per site. Data collection instruments are available (S1 File).

### Data analysis

Characteristics of AEs from GS observations were collected. Observed AE rates per site were calculated as: number of observed moderate or severe AEs divided by number of observed follow-up reviews. Ninety-fifth percentile confidence intervals (CIs) were calculated using Wilson estimates. ZAZIC program implementation standardized MC data reporting and initiated data quality audits in October 2014 [23]. We, therefore, applied the observed AE rate in each site to the number of MCs performed from October, 2014 to September, 2017 to estimate the number of AEs that might have occurred if active surveillance and QA measures had been implemented during routine program implementation in those sites over those years. As AE rates for MC programs are typically calculated using the number of moderate or severe AEs divided by the number of clients with follow-up visit within 14 days[27], the values for *expected* AEs per site were multiplied by 0.95 to account for the 95% of men who reportedly adhered to at least one post-operative visit. We subtracted *reported* from *expected* AEs to determine the number of potential missed AEs during those three years (October, 2014 to September, 2017) of routine ZAZIC program implementation. All numbers were rounded to whole numbers.

### Ethics

Study data were collected for the purpose of ZAZIC program quality improvement. Data were collected and entered with program identifiers into an aggregate database used exclusively for program monitoring. No individual identifiers were collected or used. The study received a Non-Research Determination from the University of Washington, Seattle, USA and was approved as program evaluation from the Centers of Disease Control and Prevention (CDC) Center for Global Health Associate Director for Science.

## Results

### Prospective tandem reviews

The expected observed tandem reviews were 600; 585 (97.5%) reviews were performed (Table 1). Of the 585 clients, twenty-seven were identified with AEs (4.6%) across all sites: 6 were severe AEs (22%) and 21 (78%) moderate AEs. The observed AE rates ranged from 1.0–8.0%, with an average AE rate of 4.6%. Of all observed AEs, 17 (63.0%) occurred within 7 days post-operative. The ages of those observed with an AE ranged from 10–26 years, with an average age of 11.9 years. Of the 27 AEs observed, 22 (81%) were ages 14 and under.

**Table 1:** Characteristics of the 27 AEs observed during tandem reviews.

Site	Days of GS observation	Total Reviews	Observed AEs				Observed AE rate
			AEs	Client Age (years)	AE severity	AE timing and Type	
Site 1	9	95	5	10	Moderate	B-IN	5.26%
				15	Moderate	C-IN	
				10	Moderate	B-IN	
				13	Moderate	C-IN	
				10	Moderate	B-IN	
Site 2	7	91	4	11	Moderate	B-BL	4.39%
				13	Moderate	B-IN	
				10	Moderate	C-IN	
				14	Moderate	B-IN	
Site 3	9	99	4	26	Moderate	C-IN	4.04%
				13	Moderate	B-IN	
				15	Moderate	C-IN	
				15	Severe	B-IN	
Site 4	6	100	8	10	Moderate	C-IN	8.00%
				10	Severe	B-IN	
				13	Moderate	C-IN	
				16	Severe	B-OA	
				11	Severe	B-IN	
				10	Moderate	B-IN	
				11	Moderate	B-IN	
				11	Moderate	C-IN	
Site 5	6	100	6	11	Severe	C-IN	5.0%
				10	Moderate	B-IN	
				10	Moderate	B-IN	
				10	Moderate	B-IN	
				11	Severe	B-IN	
Site 6	5	100	1	12	Moderate	C-IN	1.0%

Timing of AE: B = AE occurred within 7 days post op, C = AE occurred after 7 days post-operative. Type of AE: IN-Infection, BL- Bleeding, OA- Swelling with Hematoma

### Retrospective record review

The numbers of reported AEs should be consistent across all reporting forms. AEs were not recorded nor reported across all forms consistently (Table 2). Only one site, Site 5, had consistent AE reporting across records: they reported zero AEs during the 3-month period reviewed.

**Table 2:** Retrospective AEs found during AE form review.

	Number of Retrospective AEs Documented, Oct-Dec 2016					
Site	MC Register	MC CIF	MoHCCAE forms	In/Out patient	MRF	DHIS2
1	0	1	0	0	0	1
2	1	1	1	1	1	0
3	0	1	1	2	0	0
4	0	0	1	0	1	0
5	0	0	0	0	0	0
6	5	3	2	4	5	0

CIF-Client Intake Form, MRF- Monthly Return Form, DHIS2- District Health Information System 2.

### Estimating unidentified AEs

AE rates from the prospective, tandem reviews (observed) were higher than those documented from retrospective data (reported in DHIS2) (Table 3). Applying the observed AE rates to 95% of the previous MCs at the six sites as an estimation of unreported AEs, it is possible that 3,431 AEs (95% CI: 1,483–8,070) could have been missed in those 6 sites over three years of routine ZAZIC program implementation. Pooling the overall MC output from the 6 sites, and using the average AE rate of 4.62%, a slightly more conservative 3,032 AEs (95% CI: 2,044–4,419) may have been unidentified in those same sites between October, 2014 and September, 2017.

**Table 3:** Potential missed adverse events in 6 study sites: October 2014- September 2017.

	Reported			Observed AE rate (c)	Expected AEs** (d)	Potential unidentified AEs (d-a)
Site #	AEs* (a)	MCs (b)	AE rate			
1	10	14707	0.072%	5.26% (2.27–11.7)	735 (317–1,634)	725 (314–1,701)
2	22	13892	0.167%	4.39% (1.72–10.8)	580 (227–1,425)	558 (205–1,403)
3	30	8174	0.386%	4.04% (1.58–9.93)	314 (123–771)	284 (93–741)
4	31	17908	0.182%	8.00% (4.10–15.0)	1361 (697–2,552)	1330 (666–2,521)
5	25	11242	0.234%	5.00% (2.15–11.2)	534 (230–1,196)	509 (205–1,171)
6	39	6727	0.610%	1.00% (0.002–5.45)	64 (12–349)	25 (0–310)
Sum	157	72650	0.227%	4.62% (3.19–6.63)	3,588 (1,606–7,927)	3,431 (1,483–8,070)
Pooled	157	72650		4.62% (3.19–6.63)	3,189 (2,201–4,576)	3,032 (2,044–4,419)

*Includes only moderate and severe AEs. Observed AE rates from Table 1. Expected AEs per site (d) was calculated by multiplying (0.95) the number of MCs reported over the passive surveillance period (b) by the observed active surveillance AE rate (c) to estimate the AEs that may have actually occurred over the 2014–2017 reporting period. Potential unidentified AEs were calculated by subtracting reported AEs from expected AEs per site.

**As only 95% of men adhered to follow-up visits, the values for expected AEs per site (d) were multiplied by 0.95. Sum: Cumulative reported totals across the 6 sites; average observed AE rates across sites; total expected and unidentified AEs calculated by summing data from sites 1–6. Pooled: Calculated using the overall average observed AE rate (4.62%) multiplied by the total reported MCs (72,650). 95% CIs are presented in parentheses.

### Results from brief site questionnaires

Several findings emerged from the brief reports. First, due to transport challenges and proximity to clients’ location, site staff noted that most follow ups were completed by RHC nurses who remain in the community and not the MC team coming from the district hospital. RHC nurses are trained in wound care and MC reporting. Transportation and distances were also noted as reasons for men to miss routine follow-up visits. In lieu of returning for in-person clinical assessments, clinicians noted that some men appear to provide considerable self-care, removing their post-operative bandages themselves or consulting with their circumcised friends to judge their healing progress. As some schools did not allow all post-operative reviews to be conducted on-site during school hours, site teams revealed that some school-age clients also removed bandages on their own or judged healing among themselves to avoid school absences. Moreover, some follow-up was conducted by phone but not documented. Clinic staff did not offer explanations for the differences between reported and actual follow-up rates.

Second, sites appeared concerned with ensuring client safety. For example, if a RHC nurse identified a client with an AE that they could not manage, the static site MC team was consulted by telephone, the static MC team returned, or the client was sent to district hospital. Also, to improve on AE reviews, sites developed supplemental, innovative proactive policies to ensure safe healing. Site 5 scheduled an additional follow-up visit on day 21 for all clients who experienced an AE to ensure good progress towards complete healing while sites 1, 2 and 5 offered clients underwear to aid proper wound care. They also used informal snowball practices to help ensure adherence to follow-up visits. For example, if the site teams knew they had 20 reviews to do, but only 10 clients returned, they would engage the help of those who returned to find the missing clients, thereby increasing adherence to reviews.

Lastly, despite training in MC forms and reporting, documentation gaps for follow-ups and AE management were apparent, creating delays or disconnects in reporting outreach client data to static aggregation efforts. GS also documented lack of MC data collection tools, inadequate training for both post-operative reviews and AE identification, and lack of knowledge on correct AE reporting in five of six static sites. In the absence of MoHCC MC reporting forms in outreach settings, RHC clinicians completed follow-up visits using paper tally sheets or other ad hoc methods. MoHCC teams may also not bring MC forms to conduct outreach reviews or outreach forms may not be transferred to static sites, further leading to discrepancies in where, or if, AEs are reported. Several sites also mentioned that even if MC clients are managed correctly in RHCs, reconciliation of RHC and static site data is not routine, contributing to poor data quality that may affect both static site and DHIS2 data. Clinicians also reported use of antibiotics for clients with infections; however, as they felt confident in client management, they neglected to document the AEs, leading to further underreporting.

## Discussion

ZAZIC implemented a mixed-method, quality assurance activity in 6 purposively selected, high-volume MC sites. In contrast to reported moderate/severe AE rates ranging from 0.1%-0.6%, AE rates of 1.0–8.0% were observed through prospective, tandem, post-operative MC reviews. Retrospective record review and site interviews confirm AE data discrepancies and weaknesses in AE reporting and documentation. Although these findings are not generalizable nor definitive, they are highly suggestive that actual AE rates are higher than reported AE rates, decreasing confidence in the reliability and validity of routine AE identification and reporting. It is unlikely that this phenomenon is unique to the study sites or to ZAZIC. Significant underreporting of AEs is likely in other MC programs at scale. Despite raising concerns, there was no evidence of missed severe AEs resulting in permanent impairment or morbidity. We discuss several lessons learned and next steps to help ensure continuous quality improvement for both data quality and patient safety.

Primarily, AE underreporting appears attributable to gaps in AE documentation. Although ZAZIC data quality audits show evidence of improvements in correct and complete data collection in static sites [23], there are several likely pathways that reduce complete AE documentation. First, AEs may be treated correctly, but not reported through routine program channels. AEs treated at hospital outpatient departments or within networks of private healthcare providers are likely not reported to the MC teams. Second, although there is a standard MoHCC AE reporting form, this is not well decentralized or consistently used. Instead, AE information is reported on, and aggregated from, multiple, duplicative MoHCC and ZAZIC data collection tools that create a burden for providers. Lastly, there are weaknesses in data flow. There is only ad hoc flow of MC data from RHCs to static sites. Implementation of a single, MoHCC/ZAZIC AE reporting form for use at all points of client interaction and one channel of AE reporting could greatly reduce bottlenecks and delays.

Second, clients may not attend reviews, leading to missed AE identification. The indicator of “at least 1 follow-up visit within 14 days of MC” is reported to calculate the follow-up rate on the MRF. During site attachments, GS reported that clients’ spontaneous follow-up appeared lower than the reported 95%, echoing previous studies finding challenges in adherence to post-operative visits [14, 33, 34]. On the client side, in all MC settings, men were counselled on the healing process and where to seek care if needed. Therefore, men healing without complication may self-assess and not come for follow ups. On the healthcare worker side, shortages in human resources compound the long distances between clients and care settings especially in outreach settings. In this study, observed routine follow-up visits would largely not have occurred, or not occurred on time, in the absence of vehicle support. As a result, RHC nurses likely review only those who proactively return for follow-up at the clinic, prioritizing vehicle use for those identified by family or friends with suspected AEs. Also, it is often not feasible for MC teams, themselves, to return. Therefore, in ZAZIC’s integrated program, MC teams work jointly with RHC teams to provide follow-up coverage and care. It is possible that outreach clinicians pre-fill registers with expected follow-up visit dates, counting those aspirational dates as actual visits. In outreach settings, MC clinicians may also tell patients to seek follow-up at their local RHCs, and assume that they did, without verification. However, it is clear that all ZAZIC sites require closer supervision and more frequent QA activities to verify that all MC clients in their catchment area receive timely follow-up or document missed visits appropriately.

Lastly, ZAZIC needs to improve AE monitoring among adolescents. In our tandem reviews, 80% of observed AEs were among boys ages 14 and under. Among ZAZIC clients, overall, younger boys ages 10–14 represent the majority of ZAZIC program MCs. Although our previous research showed that these young clients are not more likely to have an AE, they are 3 times more likely to have infections as compared to their older peers [13]. Sites should distribute wound care instructions for clients and guardians, yet, most observed sites did not have wound care materials, potentially leaving parents unaware of proper wound care or the importance of routine post-operative reviews. Some guardians may also prefer to manage AEs at home. However, adolescent clients were observed with mild, moderate or severe AEs due to poor hygiene, including being unbathed, wearing dirty underwear, or using dirty cloth or string to elevate the penis. It appears difficult for younger clients to maintain clean wounds [35, 36]. Widespread misperceptions of how to speed healing through traditional herbal remedies or use of hypertonic saline also encourage infection [37, 38]. In response, ZAZIC should conduct additional community and school dialogues with parents and youth before MC campaigns and complement these activities with revised wound care instructions tailored for those with lower literacy [39].

Several reassuring practices emerged from this activity. First, the majority of clients with scheduled follow-up visits were located and MC verification completed as part of the review. Second, despite documentation weaknesses, AEs were properly identified and managed at all care settings, both static and outreach, suggesting that providers are providing quality care for MC clients. Also, few discrepancies in AE identification or severity grading were found during tandem reviews, demonstrating that MoHCC MC clinicians are well skilled. Lastly, all MoHCC clinicians identified, completed, and reported AEs according to MOHCC standard reporting guidelines when observed, confirming correct knowledge of AE protocols and policies.

This study had several limitations. First, it required additional financial, transport, and human resources over routine program monitoring. Second, only 6 of 21 sites were assessed on a limited number of days and all service providers were not observed. Therefore, findings may have differed if other clients, clinicians, or calendar days, were included. The GS vehicle enabled active follow up that otherwise may not have occurred; therefore, it is possible that findings may have differed from observations of clients who were able to attend visits without vehicle support. Moreover, post-operative follow-up visits were reported at approximately 95% across all sites; however, sub-optimal client attendance at routine post-operative visits was observed and noted in GS discussions with site teams. This suggests discrepancies between reported and actual follow-up visits. GS did not record the number of times they failed to locate a client; therefore, we cannot report the percent of clients reviewed as compared to those scheduled. In subsequent investigations, GS should conduct a more formal assessment of actual versus reported adherence to routine follow-up visits. Additionally, as GS did not observe 100 consecutive reviews, it is possible that more skilled clinicians were assigned to the GS on subsequent visits or that the observed clinicians improved as a result of the tandem review process. However, this would not alter the overall number of AEs identified, reducing the magnitude of this potential bias on results. Moreover, the observed AE rate from tandem reviews at each site was applied as a constant value to previous MCs in those same sites over a large time period to estimate potential missed AEs (Table 3, column (d)). Many changes likely took place between 2014 and 2017 that could have influenced AE rates. However, as both the MC programs and clinicians likely improved over time, resulting in lower AE rates by the 2017 observation period, we feel confident that there were likely a large number of unidentified or unreported AEs over those previous years. Lastly, AE definitions may vary over time, setting, or between countries, limiting quantitative comparison of AE rates between programs or over time. However, as there are few prospective studies of active surveillance for AEs in programs running at scale, these findings are highly relevant for others implementing MC programs in the region.

## Conclusions

Reported AE rates collected through passive surveillance appear low. In contrast, the active surveillance employed through this QA study found that observed AE rates are considerably higher while record review found that AE data quality is sub-optimal. It is unlikely that this phenomenon is unique. Although the results may not be definitive nor generalizable, they require follow-up action. ZAZIC promotes quality assurance and patient safety as critical components of its MC implementation. Therefore, to meet the study objectives of strengthening AE documentation and increase confidence in AE data quality, there were several quality assurance activities implemented to address the study findings.

First, in early 2018, ZAZIC launched a Quality Improvement Task Force to conduct continuous quality improvement activities including spot audits and data quality reviews focused on AEs. Second, ZAZIC and the MoHCC reinforced clear expectations with regard to recognition and reporting of AEs by developing a single, standardized AE reporting tool for implementation at static, outreach, and outpatient care settings where clients may seek care. In combination with improved decentralization of, and training on, the MoHCC AE reporting form, this tool aims to help ensure that AE reports are integrated consistently into DHIS2. Third, ZAZIC and MoHCC continue to reassure service providers that reporting AEs is a sign of quality programing and does not result in punishment. Fourth, in rural areas, ZAZIC conducts intensive training in post-operative MC reviews, AE surveillance, and AE reporting for RHC nurses to further strengthen AE management, data collection and reporting. Fifth, as additional nurses and vehicles, especially in rural areas, are needed to ensure patient follow-up adherence and maintain program quality while expanding coverage, ZAZIC continuously trains healthcare workers in AE recognition and further expanded its vehicle fleet to support MoHCC operations. Lastly, enhanced risk reduction strategies are still needed for younger clients within home and school settings. In response, ZAZIC revised wound care instructions for clients and care givers, distributing them widely to sites beginning in fall, 2018. Overall, continuous monitoring of, and reporting on, client safety would strengthen MC program quality and facilitate the establishment of sustainable, safe MC programs at scale.

## Supporting information

S1 File Data collection instruments. (PDF)

## References

[1] World Health Organization. Joint strategic action framework to accelerate the scale-up of voluntary medical male circumcision for HIV prevention in Eastern and Southern Africa (2012–2016). Geneva: UNAIDS 2011.

[2] Grund JM. Notes from the field: tetanus cases after voluntary medical male circumcision for HIV prevention—Eastern and Southern Africa, 2012–2015. MMWR Morbidity and mortality weekly report. 2016;65. 10.15585/mmwr.mm6502a5 26797167

[3] Auvert B, Taljaard D, Lagarde E, Sobngwi-Tambekou J, Sitta R, Puren A. Randomized, controlled intervention trial of male circumcision for reduction of HIV infection risk: the ANRS 1265 Trial. PLoS medicine. 2005;2(11):e298 10.1371/journal.pmed.0020298 16231970 PMC1262556

[4] Bailey RC, Moses S, Parker CB, Agot K, Maclean I, Krieger JN, et al Male circumcision for HIV prevention in young men in Kisumu, Kenya: a randomised controlled trial. Lancet. 2007;369(9562):643–56. Epub 2007/02/27. 10.1016/S0140-6736(07)60312-2 . 17321310

[5] Gray RH, Kigozi G, Serwadda D, Makumbi F, Watya S, Nalugoda F, et al Male circumcision for HIV prevention in men in Rakai, Uganda: a randomised trial. The Lancet. 2007;369(9562):657–66. 10.1016/S0140-6736(07)60313-4 17321311

[6] World Health Organization. WHO technical advisory group on innovations in male circumcision: evaluation of two adult devices: Meeting report. Geneva, Switzerland: WHO, January, 2013 8/10/2015. Report No.

[7] Byabagambi J, Kigonya A, Lawino A, Ssensamba JT, Twinomugisha A, Karamagi-Nkolo E, et al. A guide to improving the quality of safe male circumcision in Uganda 2015 [cited 2016 August 12]. Available from: https://www.usaidassist.org/sites/assist/files/uganda_guide_to_improving_the_quality_of_smc_a4_feb2015_ada.pdf

[8] President’s Emergency Plan for AIDS Relief. PEPFAR's best practices for voluntary medical male circumcision site operations: A service guide for site operations. 2013 May 2014. Available from: http://www.malecircumcision.org/resources/documents/VMMC%20Best%20Practices03.04.2013_web.pdf

[9] President’s Emergency Plan for AIDS Relief. PEPFAR's best practices for voluntary medical male circumcision site operations: A service guide for site operations. Managing, monitoring, and reporting VMMC adverse events [Internet]. 2017. Available from: https://www.malecircumcision.org/sites/default/files/document_library/2017.9.26_ch7_vmmc-site-ops.pdf

[10] Reed J, Grund J, Liu Y, Mwandi Z, Howard AA, McNairy ML, et al Evaluation of loss-to-follow-up and post-operative adverse events in a voluntary medical male circumcision program in Nyanza Province, Kenya. Journal of acquired immune deficiency syndromes (1999). 2015. 10.1097/QAI.0000000000000535 25942466

[11] Herman-Roloff A, Bailey RC, Agot K. Factors associated with the safety of voluntary medical male circumcision in Nyanza province, Kenya. Bulletin of the World Health Organization. 2012;90(10):773–81. Epub 2012/10/31. 10.2471/BLT.12.106112 23109745 PMC3471059

[12] Bailey RC, Egesah O, Rosenberg S. Male circumcision for HIV prevention: a prospective study of complications in clinical and traditional settings in Bungoma, Kenya. Bulletin of the World Health Organization. 2008;86(9):669–77. Epub 2008/09/18. 10.2471/BLT.08.051482 18797642 PMC2649497

[13] Bochner A, Feldacker C, M Makunike-Chikwinya B, Holec M, Murenje V, Stepaniak A, et al Adverse event profile of a mature voluntary medical male circumcision progra mme performing PrePex and surgical procedures in Zimbabwe. Journal of the International AIDS Society. 2017;(20:21394). Epub 21 February 2017. 10.7448/IAS.20.1.21394 PMC5467584 28362066

[14] Lissouba P, Taljaard D, Rech D, Doyle S, Shabangu D, Nhlapo C, et al A model for the roll-out of comprehensive adult male circumcision services in African low-income settings of high HIV incidence: the ANRS 12126 Bophelo Pele Project. PLoS medicine. 2010;7(7):e1000309 10.1371/journal.pmed.1000309 20652013 PMC2907271

[15] Maponga BA, Chirundu D, Shambira G, Gombe NT, Tshimanga M, Bangure D. Evaluation of the notifiable diseases surveillance system in sanyati district, Zimbabwe, 2010–2011. The Pan African medical journal. 2014;19. 10.11604/pamj.2014.19.278.5202 PMC4391897 25870733

[16] World Health Organization. Country health information systems: a review of the current situation and trends: World Health Organization; 2011.

[17] World Health Organization. Voluntary medical male circumcision for HIV prevention in 14 priority countries in Eastern and Southern Africa. Geneva: WHO, 2017 July, 2017. Report No.

[18] Ministry of Health and Child Care Zimbabwe. Accelerated strategic and operational plan 2014–2018: Voluntary Medical Male Circumcision. Harare, Zimbabwe: 2014.

[19] Byabagambi J, Marks P, Megere H, Karamagi E, Byakika S, Opio A, et al Improving the quality of voluntary medical male circumcision through use of the continuous quality improvement approach: a pilot in 30 PEPFAR-supported sites in Uganda. PloS one. 2015;10(7):e0133369 10.1371/journal.pone.0133369 26207986 PMC4514600

[20] Ledikwe JH, Grignon J, Lebelonyane R, Ludick S, Matshediso E, Sento BW, et al Improving the quality of health information: a qualitative assessment of data management and reporting systems in Botswana. Health research policy and systems. 2014;12(1):7. 24479822 10.1186/1478-4505-12-7 PMC3910237

[21] Kohler PK, Namate D, Barnhart S, Chimbwandira F, Tippet-Barr BA, Perdue T, et al Classification and rates of adverse events in a Malawi male circumcision program: impact of quality improvement training. BMC Health Serv Res. 2016;16(1):61 Epub 2016/02/19. 10.1186/s12913-016-1305-x 26888178 PMC4758015

[22] Adamson PC, Tafuma TA, Davis SM, Xaba S, Herman-Roloff A. A systems-based assessment of the PrePex device adverse events active surveillance system in Zimbabwe. PloS one. 2017;12(12):e0190055 10.1371/journal.pone.0190055 29272320 PMC5741245

[23] Xiao Y, Bochner A, Makunike B, Holec M, Xaba S, Tshimanga M, et al Challenges in data quality: the influence of data quality assessments on data availability and completeness in a voluntary medical male circumcision programme in Zimbabwe. BMJ open. 2017;7(1):e013562 10.1136/bmjopen-2016-013562 28132009 PMC5278271

[24] Feldacker C, Makunike-Chikwinya B, Holec M, Bochner AF, Stepaniak A, Nyanga R, et al Implementing voluntary medical male circumcision using an innovative, integrated, health systems approach: experiences from 21 districts in Zimbabwe. Global health action. 2018;11(1):1414997 10.1080/16549716.2017.1414997 29322867 PMC5769777

[25] Marongwe P, Feldacker C, Gonouya P, Madoda T, Tshimanga M, Balachandra S, et al. Trust but Verify: Is there a role for active surveillance in monitoring AEs in large-scale VMMC programs? Poster presented at: 22nd International AIDS Conference (AIDS 2018); 23–27 July 2018; Amsterdam, Netherlands Amsterdam, Netherlands 2018.

[26] Feldacker C, Bochner AF, Murenje V, Makunike-Chikwinya B, Holec M, Xaba S, et al Timing of adverse events among voluntary medical male circumcision clients: Implications from routine service delivery in Zimbabwe. PloS one. 2018;13(9):e0203292 10.1371/journal.pone.0203292 30192816 PMC6128519

[27] President’s Emergency Plan for AIDS Relief. PEPFAR monitoring, evaluation, and reporting indicator reference guide. March 2015 ed. Washington DC: PEPFAR; 2015.

[28] World Health Organization. Manual for male circumcision under local anaesthesia and HIV prevention services for adolescent boys and men. Geneva: WHO [Internet]. 2018 Feburary 1, 2019 [cited 2019. Available from: https://www.who.int/hiv/pub/malecircumcision/male-circumcision-guide-2018/en/

[29] Ministry of Health and Child Care Zimbabwe. Standard operating procedures for voluntary medical male circumcision. In: Zimbabwe MoHCC, editor. Harare: MoHCC; 2015.

[30] World Health Organization. WHO surgical safety checklist 2009 [cited 2019 February 6,]. Available from: https://www.who.int/patientsafety/safesurgery/checklist/en/

[31] World Health Organization. Considerations for implementing models for optimizing the volume and efficiency of male circumcision services2010 [cited 2014 June 1]; Field testing edition: February 2010 Available from: http://www.malecircumcision.org/programs/documents/mc_MOVE_2010_web.pdf

[32] World Health Organization. Guideline on the use of devices for adult male circumcision for HIV prevention2013 [cited 2017 March 2]. Available from: http://apps.who.int/iris/bitstream/10665/93178/1/9789241506267_eng.pdf?ua=1

[33] Phili R, Abdool-Karim Q, Ngesa O. Low adverse event rates following voluntary medical male circumcision in a high HIV disease burden public sector prevention programme in South Africa. Journal of the International AIDS Society. 2014;17(1). 10.7448/IAS.17.1.19275 PMC4236629 25406951

[34] Wynn A, Bristow CC, Ross D, Schenker I, Klausner JD. A program evaluation report of a rapid scale-up of a high-volume medical male circumcision site, KwaZulu-Natal, South Africa, 2010–2013. BMC health services research. 2015;15(1):235. 26084777 10.1186/s12913-015-0904-2 PMC4472417

[35] Mavhu W, Hatzold K, Dam KH, Kaufman MR, Patel EU, Van Lith LM, et al Adolescent wound-care self-efficacy and practices after voluntary medical male circumcision—a multicountry assessment. Clinical Infectious Diseases. 2018;66(suppl_3):S229–S35. 10.1093/cid/cix953 29617777 PMC5888964

[36] Tobian AA, Dam KH, Van Lith LM, Hatzold K, Marcell AV, Mavhu W, et al Providers’ perceptions and training needs for counseling adolescents undergoing voluntary medical male circumcision. Clinical Infectious Diseases. 2018;66(suppl_3):S198–S204. 10.1093/cid/cix1036 29617772 PMC5888966

[37] World Health Organization. Traditional male circumcision among young people: a public health perspective in the context of HIV prevention. 2009.

[38] ZAZIC. How to take care of yourself after surgical male circumcision. March 2017 ed. Harare, Zimbabwe: ZAZIC; 2017.

[39] Lane C, Bailey RC, Luo C, Parks N. Adolescent male circumcision for HIV prevention in high priority countries: opportunities for improvement. clinical infectious diseases. 2018;66(suppl_3):S161–S5. 10.1093/cid/cix950 29617774 PMC5888994

