# Supplementary material for: Trust but verify: Is there a role for active surveillance in monitoring adverse events in Zimbabwe’s large-scale male circumcision program?
Source: PLoS One. 2019 Jun 10;14(6):e0218137. doi: 10.1371/journal.pone.0218137 (PMC6557516; doi:10.1371/journal.pone.0218137)
Supplement: S1 File — (PDF) [file pone.0218137.s001.pdf]

Prospective Activity: AE Review Form – client VMMC follow-up review

Site name_______________________________ Date___________________________Site type: Outreach Mobile Static

Reviewer_______________________ Dates of in-person reviews observed________________________ Cadre of person observed: nurse doctor

VMMC follow-up reviews	Client order																								
	1	2	3	4	5	6	7	8	9	10	11	12	13	14	15	16	17	18	19	20	21	22	23	24	25
**Client review characteristics**																									
Review Day	2<br>7<br>14<br>___	2<br>7<br>14<br>___	2<br>7<br>14<br>___	2<br>7<br>14<br>___	2<br>7<br>14<br>___	2<br>7<br>14<br>___	2<br>7<br>14<br>___	2<br>7<br>14<br>___	2<br>7<br>14<br>___	2<br>7<br>14<br>___	2<br>7<br>14<br>___	2<br>7<br>14<br>___	2<br>7<br>14<br>___	2<br>7<br>14<br>___	2<br>7<br>14<br>___	2<br>7<br>14<br>___	2<br>7<br>14<br>___	2<br>7<br>14<br>___	2<br>7<br>14<br>___	2<br>7<br>14<br>___	2<br>7<br>14<br>___	2<br>7<br>14<br>___	2<br>7<br>14<br>___	2<br>7<br>14<br>___	2<br>7<br>14<br>___
AE identified by site staff	Y<br>N																								
Actual AE present	Y<br>N																								
AE type identified correctly	Y<br>N																								
AE severity identified correctly	Y<br>N																								
AE reviewed with staff	Y<br>N																								

TOTAL NUMBER OF AEs IDENTIFIED BY SITE STAFF:_________________________TOTAL NUMBER OF AEs IDENTIFIED BY ZAZIC STAFF____________________

Notes____
____
____

Site team QA representative signature____

<u>Retrospective</u> AE Review Form – Register Data Review - July-December 2016

Site name______________________________ Date__________________________Site type: Outreach Mobile Static

Reviewer______________________ Month(s) of reviewed data______________ Register pages reviewed:_______________

Site team QA representative signature____

VMMC procedure characteristics	VMMC register **PAGE** reviewed (check if complete. Leave BLANK if incomplete or incorrect)																								
	1	2	3	4	5	6	7	8	9	10	11	12	13	14	15	16	17	18	19	20	21	22	23	24	25
**VMMC Register**																									
Adverse event section complete - Day of Procedure (severity + code)																									
1st post-procedure review AE section complete (Day 2 or Day 7) (severity + code)																									
2nd post-procedure review AE section complete (Day 7 or Day 14) (severity + code)																									
3rd post-procedure review AE section complete (Day 42 or Day 49) (severity + code)																									
Additional post-procedure review AE section complete (if applicable) (severity + code)																									
Comments completed – if applicable																									

<u>IN THE PAST 3 MONTHS:</u>

TOTAL NUMBER OF AEs IDENTIFIED FROM DATA:___________ TOTAL NUMBER OF AEs REPORTED ON MRF TO PARTNER:__________TOTAL NUMBER OF AEs IN DHIS2:__________:__________

Notes____

____

<u>Retrospective</u> AE Review Form – CIF Data Review - July-December 2016

Site name_______________________________ Date___________________________Site type: Outreach Mobile Static

Reviewer_______________________ Month(s) of reviewed data______________ **CIF file numbers reviewed**:_______________

Site team QA representative signature____

VMMC procedure characteristics	CIF reviewed (check if complete. Leave BLANK if incomplete or incorrect)																								
	1	2	3	4	5	6	7	8	9	10	11	12	13	14	15	16	17	18	19	20	21	22	23	24	25
**CIF**																									
Adverse event section complete - Day of Procedure (severity + code)																									
1st post-procedure review AE section complete (Day 2 or Day 7) (severity + code)																									
2nd post-procedure review AE section complete (Day 7 or Day 14) (severity + code)																									
3rd post-procedure review AE section complete (Day 42 or Day 49) (severity + code)																									
Additional post-procedure review AE section complete (if applicable) (severity + code)																									
Comments completed – if applicable																									

<u>IN THE PAST 3 MONTHS:</u>

TOTAL NUMBER OF AEs IDENTIFIED FROM DATA:___________ TOTAL NUMBER OF AEs REPORTED ON MRF TO PARTNER:__________TOTAL NUMBER OF AEs IN DHIS2:__________

Notes____

____

Retrospective AE Review Form – AE Log Review - July-December 2016

Site name_______________________________ Date___________________________Site type: Outreach Mobile Static

Reviewer______________________ Month(s) of AE Log reviewed:____

Site team QA representative signature____

VMMC procedure characteristics	AE Log Month reviewed (check if complete. Leave BLANK if incomplete or incorrect)																								
	1	2	3	4	5	6	7	8	9	10	11	12	13	14	15	16	17	18	19	20	21	22	23	24	25
**1. AE Log**																									
Any AE reported?																									
If yes, take photo of each report/line with client name/number covered																									

IN THE PAST 3 MONTHS:

TOTAL NUMBER OF AEs IDENTIFIED FROM DATA:___________ TOTAL NUMBER OF AEs REPORTED ON MFR TO PARTNER:__________TOTAL NUMBER OF AEs IN DHIS2:__________

Notes____
____
____

Details on AE on-site management

1. Who does AE documentation at your site?
2. What forms do you use to document AEs?
3. How do you report AEs at your facility and what are the reporting timelines?
4. Do you have challenges in reporting AEs?
5. Who reports/compiles/completes AEs on the MRFs?
6. Do you think the team has been picking up and reporting AEs. If not what may be the causes
7. Does your site face challenges in doing reviews? Probe for transport, Staffing and AE review schedule challenges
8. Can you describe your facility's AE review system?
